# Mayaro Virus Infection in Amazonia: A Multimodel Inference Approach to Risk Factor Assessment

**DOI:** 10.1371/journal.pntd.0001846

**Published:** 2012-10-11

**Authors:** Fernando Abad-Franch, Gustavo H. Grimmer, Vanessa S. de Paula, Luiz T. M. Figueiredo, Wornei S. M. Braga, Sérgio L. B. Luz

**Affiliations:** 1 Instituto Leônidas e Maria Deane – Fiocruz Amazônia, Manaus, Amazonas, Brazil; 2 Instituto Oswaldo Cruz – Fiocruz, Rio de Janeiro, Brazil; 3 Faculdade de Medicina de Ribeirão Preto, Universidade de São Paulo, Ribeirão Preto, São Paulo, Brazil; 4 Fundação de Medicina Tropical do Estado do Amazonas, Manaus, Amazonas, Brazil; University of Texas Medical Branch, United States of America

## Abstract

**Background:**

Arboviral diseases are major global public health threats. Yet, our understanding of infection risk factors is, with a few exceptions, considerably limited. A crucial shortcoming is the widespread use of analytical methods generally not suited for observational data – particularly null hypothesis-testing (NHT) and step-wise regression (SWR). Using Mayaro virus (MAYV) as a case study, here we compare information theory-based multimodel inference (MMI) with conventional analyses for arboviral infection risk factor assessment.

**Methodology/Principal Findings:**

A cross-sectional survey of anti-MAYV antibodies revealed 44% prevalence (*n* = 270 subjects) in a central Amazon rural settlement. NHT suggested that residents of village-like household clusters and those using closed toilet/latrines were at higher risk, while living in non-village-like areas, using bednets, and owning fowl, pigs or dogs were protective. The “minimum adequate” SWR model retained only residence area and bednet use. Using MMI, we identified relevant covariates, quantified their relative importance, and estimated effect-sizes (*β*±SE) on which to base inference. Residence area (*β*_Village_ = 2.93±0.41; *β*_Upland_ = −0.56±0.33, *β*_Riverbanks_ = −2.37±0.55) and bednet use (*β* = −0.95±0.28) were the most important factors, followed by crop-plot ownership (*β* = 0.39±0.22) and regular use of a closed toilet/latrine (*β* = 0.19±0.13); domestic animals had insignificant protective effects and were relatively unimportant. The SWR model ranked fifth among the 128 models in the final MMI set.

**Conclusions/Significance:**

Our analyses illustrate how MMI can enhance inference on infection risk factors when compared with NHT or SWR. MMI indicates that forest crop-plot workers are likely exposed to typical MAYV cycles maintained by diurnal, forest dwelling vectors; however, MAYV might also be circulating in nocturnal, domestic-peridomestic cycles in village-like areas. This suggests either a vector shift (synanthropic mosquitoes vectoring MAYV) or a habitat/habits shift (classical MAYV vectors adapting to densely populated landscapes and nocturnal biting); any such ecological/adaptive novelty could increase the likelihood of MAYV emergence in Amazonia.

## Introduction

Arboviral infections are a major global public health concern; dengue is the most widespread, but other viruses in the families *Flaviviridae*, *Togaviridae* or *Bunyaviridae* are also emerging worldwide [1]–[3]. A solid understanding of the epidemiology of emerging arboviral diseases is crucial for the development and operation of functional control/surveillance systems [2], [4]. However, except for dengue virus (e.g., [5]–[7]) and a few other arboviruses (e.g., [8]–[10]), risk factors for infection remain poorly understood.

Apart from overall neglect resulting in fewer epidemiological studies than would be needed [11], poor data analysis and presentation in published reports also hinder our understanding of arboviral infection risk factors. On the one hand, most reports are merely descriptive, thus precluding formal inference; on the other, infection survey data are often analyzed with inadequate statistical techniques. In particular, null hypothesis-testing (NHT) statistics and step-wise regression (SWR) analysis have been repeatedly criticized for their many drawbacks in the analysis of observational data (e.g., [12]–[17]).

Among the major practical shortcomings of NHT is the fact that p-values provide no information on the size, direction, or precision of effect estimates; such estimates, in the form of, for instance, regression slope parameters or odds ratios (with their associated standard errors and/or confidence intervals), are central to inference [12]–[17]. In addition, NHT p-values represent the probability of the observed (or more extreme) data, given the (presumably false) null hypothesis [13], [17]. As Jacob Cohen put it, this is not “what we want to know”; rather, we want to know, at least, how likely the null hypothesis is, given the data (ref. [13], p. 997). Taking this argument a little further, we aim to examine the likelihood of (or strength of evidence for) several alternative, plausible hypotheses by confronting them with empirical data [17]–[20]. In epidemiology, this is often accomplished with the aid of statistical models. Since several candidate covariates (putative risk factors and confounders) are usually examined in different combinations, model selection procedures are used to ‘retain’ only those that appear as important in a final, ‘minimum adequate model’ on which inference is then based. The most widely used of these procedures apply step-wise algorithms in which NHT-derived p-values are used to decide whether a particular covariate should be retained or dropped from the model [16]. Apart from relying on a mechanical application of p-values from multiple null hypothesis tests, step-wise procedures can lead to biased parameter estimates and disregard the variance component due to model selection uncertainty [15], [16], [18]–[20]. A framework for inference based on likelihood and information theories has been developed that avoids many of the pitfalls of NHT and SWR; it relies on comparing multiple models, representing alternative *a priori* hypotheses, based on both their fit to the data and their complexity [15]–[20]. Multimodel inference (MMI) then proceeds by examining model-averaged effect-size estimates for all the covariates of interest [15], [19], [20]. These approaches are being increasingly applied in infectious disease epidemiology (e.g., [21]–[23]), but have seldom been used for assessing emerging arboviral disease risk [8]–[10], [24]–[26].

Here, we analyze data from a cross-sectional serological survey on Mayaro virus (MAYV) infection as a case-study to illustrate how MMI can enhance arbovirus infection risk factor analyses. MAYV is an alphavirus transmitted primarily by diurnal, canopy-dwelling mosquitoes of the genus *Haemagogus* [3], [27]. It causes an acute, dengue-like febrile illness accompanied by rash and severe arthralgia that is often highly incapacitating [3], [27]–[29]. MAYV infection is a candidate for emergence as a major public health problem, much in the way it recently happened with the closely-related chikungunya virus when it adapted to urban *Aedes* mosquitoes [3], [30], [31]. However, available epidemiological evidence suggests that MAYV transmission is largely restricted to sylvatic cycles involving non-human vertebrates, with limited spillover to human hosts who make frequent use of forest habitats in tropical South America [3], [4], [27]–[29], [32]–[37]. Such a scenario implies that MAYV infection risk must be higher among human groups living or working regularly in well-preserved, forested landscapes than among those living in degraded landscapes or rarely entering forest habitats (e.g., children). Here we use MAYV serology (IgG) data to test this prediction. Furthermore, we compare the performance of NHT, SWR, and MMI at identifying and quantifying risk factors for MAYV infection in a typical central Amazon rural setting.

## Methods

### Ethics statement

This study was approved by the Fiocruz Institutional Review Board, Brazil (Protocol 384/07); all participants gave written informed consent. Laboratory procedures involving mice followed the guidelines of the Brazilian National Council for the Control of Animal Experimentation (CONCEA) and were approved by the Institutional Review Board for Animal Research of the School of Medicine, University of São Paulo at Ribeirão Preto, Brazil (Protocol 115/2008).

### Study setting

In the context of a study on infectious disease ecology in the central Brazilian Amazon, we conducted a cross-sectional serological survey (see Text S1) in a rural settlement of the agricultural frontier. The settlement (*N* = 583 inhabitants in 158 households within a *terra firme* rain forest matrix) is located ∼150 km north of Manaus (∼1°48′S; 60°19′W) and consists of two village-like household clusters (30×50 m plots for a house with courtyard) plus extensive upland and riverbank areas with scattered households (in ∼250×2000 m farm plots) and hence lower population density; old-growth forests comprise most of the ∼280 km^2^ of the settlement. Typical upland houses are located in large forest clearings used for farming, whereas most riverbank houses, which have only boat access, are located in smaller clearings within better-preserved forest. To the north and west, the settlement shares boundaries with a large indigenous reserve composed of pristine forests. Agriculture is the most important economic activity in the settlement; the main crops are banana, manioc, papaya, black beans, rice, maize, and the native *cupuaçu* (*Theobroma grandiflorum*) and *pupunha* (*Bactris gasipaes*). Most of the harvest is consumed within the community. Only a few settlers raise cattle for commercial purposes, but many families breed other domestic animals (mainly fowl and pigs) for their own use; dogs and cats are common. Forest extractivist products include timber, Brazil nuts (*Bertholletia excelsa*), and a variety of medicinal herbs. Non-commercial hunting and fishing are also relatively common.

The allochthonous dengue vectors, *Aedes aegypti* and *Ae. albopictus*, have never been collected in longitudinal mosquito studies conducted in this remote settlement. At the time of our survey, *Culex* (*Culex*) and *Cx.* (*Melanoconion*) were the dominant mosquito subgenera in the village-like clusters, although *Psorophora*, *Anopheles*, and *Coquillettidia* were also present. *Haemagogus*, *Sabethes*, *Ochlerotatus*, *Wyeomyia*, and *Trichoprosopon* were only recorded in forest sites, whereas *Aedeomyia*, *Mansonia*, and *Uranotaenia* occurred only in crop-plots; *Anopheles*, *Culex*, *Psorophora*, and *Coquillettidia* were present in all three environments (SLBL, unpublished data).

### Serology

Finger-prick bloodspots from 270 randomly selected subjects (83 households: 42 in upland, 26 in village-like, and 15 in riverbank areas) were collected onto filter paper in 2007; the sample was representative of the community and ensured 0.05 precision for an expected prevalence of 50% (required sample size, finite population correction: *n* = 232). Sera were tested for anti-MAYV IgG antibodies using an enzyme-linked immunoassay with infected cultured cells as antigenic matrix (EIA-ICC), following procedures described elsewhere [38], [39]. Briefly, *Aedes albopictus* C6/36 cells were cultured in Leibovitz L-15 medium with bovine fetal serum (Invitrogen), penicillin, and streptomycin. Cells were infected with lyophilized baby mouse infected brain tissue resuspended in PBS; cells and virus suspension were incubated for 3–4 days at 28°C. Infected and uninfected (negative control) cells were transferred to TPP tissue culture plates (Sigma-Aldrich), which were incubated at 28°C for 24 h. A formalin solution was then added (18 h at 4°C) and wells washed with PBS. Non-specific binding sites were blocked for 1 h at 37°C with skim milk (5% plus 0.05% Tween-20 in PBS). Eluted serum samples (100 µL) were then added to each well pair (one with and one without viral antigen); mouse immune ascitic fluid was used as a positive control. Plates were then incubated (1 h, 37°C) and washed; 100 µL of 1∶200 peroxidase-labeled anti-human (test wells) or anti-murine (positive control wells) IgG antibody (KPL Inc.) were added to each well. Plates were again incubated at 37°C for 1 h, washed, and 50 µL of ABTS peroxidase substrate (KPL Inc.) were added to each well; after 15 min at room temperature, enzyme activity was blocked with 50 µL H_2_SO_4_, and plates read in a spectrophotometer at 450 nm. In each plate, absorbance values (test well minus paired negative control well) were averaged (â) and the standard deviation (SD) calculated; the plate cut-off value for positivity was â+3SD.

### Covariates

Participants were interviewed for information on three groups of putative risk factors and/or confounders:

- individual-level traits: age (years), gender (male/female), and regular bednet use (yes/no);
- household-level traits: residence area within the settlement (village-like clusters, riverbanks, upland); basic sanitation (since there was no sewage system in the settlement, this covariate described whether or not there was a closed toilet/latrine in the household; yes/no); adequate solid waste disposal (yes/no; here, ‘adequate’ means that waste was eliminated from the household's surroundings, mainly by burying/burning it at a sufficiently large distance); and ownership of a crop-plot – locally known as *roça* and usually located in forest clearings (yes/no); and
- whether or not domestic fowl, dogs, cats, or pigs, which may represent bloodmeal sources for female mosquito vectors, were reared or kept near the household (yes/no for each one).

### Data analyses

We first screened the dataset for associations between anti-MAYV seropositivity and putative risk factors with NHT statistics, using either Fisher's exact tests or likelihood-ratio (LR) χ^2^ tests from bivariate logistic regression. At this stage, we also checked for correlation between potential predictor variables. If any pair of covariates proved to be highly correlated, we planned to retain only that for which a clear hypothetical relationship with MAYV transmission could be specified; however, all correlation coefficients were <0.30 (details not shown).

We then adopted the SWR approach [16] that has become the conventional standard in risk factor analysis (e.g., for arboviruses, [5], [7], [40]–[43]). Starting with a saturated, additive logistic model including all covariates for which NHT suggested a correlation (defined *a priori* as those with bivariate p≤0.10), we removed, at each step, the covariate with the largest p-value (from LR tests) until all covariates in the final, ‘minimum adequate’ model [16] had p-values<0.05.

Finally, we implemented a MMI strategy (see refs. [18]–[20] for details) including the following steps:

- fitting three subsets of models with only individual-level, only household-level, and only domestic animal covariates;
- selecting unequivocally important covariates, as quantitatively assessed by their relative importance (see below) for predicting seropositivity status in each subset;
- specifying and fitting the complete set of additive logistic regression models for the selected covariates (i.e., an all-subsets approach);
- back-checking that none of the covariates excluded after step (ii) improved the performance of the models with substantial support from the data identified in step (iii); and
- estimating weighted mean effect-sizes (see below) and the relative importance of each covariate based on the final model set.

Logistic regression models were of the simple general form
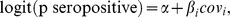
where *α* is the intercept and *β_i_* represents the effect of covariate *i* (*cov_i_*) on the (logit-scale) probability that a subject is MAYV-seropositive. Models were fit in JMP 9.0.0 (SAS Institute), with parameters estimated via maximum likelihood, and compared using Akaike's Information Criterion corrected for small sample size (AICc); AICc combines likelihood and information theories to identify, within a given set of models, those with a better compromise between fit and complexity, providing an estimate of Kullback-Leibler information loss. AICc is given by
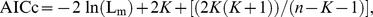
where L_m_ is the likelihood of the data given each fitted model, *K* is the number of estimable parameters in each model, and *n* is sample size.

For each model *i*, we calculated the variation in AICc relative to the best-ranking (lowest AICc) model (ΔAICc = AICc*_i_*−AICc_min_); models with ΔAICc<2 are generally taken to be substantially supported by the data. The likelihood of each model given the data was estimated as L (model | data) = exp(−ΔAICc/2); these values were then used to compute Akaike weights (denoted *w_i_*), which are normalized model likelihoods, as:
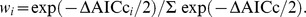
The relative importance of each covariate (denoted *w*) was estimated, within each model set, as the sum of Akaike weights over all models in which the covariate was present; covariates with *w*≤0.35 were considered unimportant. Weighted mean effect-sizes (*β*s) were estimated, for each covariate within the final model set, as the sum of model-specific effect sizes times model-specific Akaike weights. Finally, model fit was assessed using goodness-of-fit χ^2^ tests and scaled generalized *R* ^2^ values [44], with
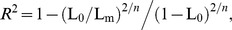
where L_0_ is the likelihood of the data given the intercept-only model, L_m_ is the likelihood of the data given the fitted model, and *n* is sample size.

## Results

### Descriptive results and null hypothesis-testing

Anti-MAYV antibodies were detected in 119 serum samples (44.1%). NHT suggested no departure from a random distribution of seropositivity in relation to sex or age; 36.8% of 19 toddlers fewer than three years old were seropositive, and there was very little variation across age classes (Table 1). Surprisingly for a virus transmitted by forest-dwelling vectors, seropositivity was strongly, positively associated with living in household-like village clusters and negatively associated with living in the better-preserved riverbank areas. Using a closed toilet/latrine also increased risk, whereas regularly sleeping under a bednet and owning/rearing fowl, pigs, or dogs were apparently protective. No association was detected between seropositivity and owning cats, owning crop-plots, or whether solid waste disposal at the household level was or was not adequate (Table 1).

**Table 1:** Mayaro virus seroprevalence in a rural Amazonian settlement: descriptive and bivariate null hypothesis-testing statistics.

Variable	Levels	Seropositive		Total	% ⊕	OR (95%CI)	Test (d.f.)	p-value
		Yes	No					
Overall	-	119	151	270	44.1	-	-	-
Sex	Female	63	83	146	43.2	1 (reference)		
	Male	56	68	124	45.2	1.08 (0.67–1.76)	FET	0.810
Age	1-yr increment	-	-	-	-	1.004 (0.99–1.02)	LR χ^2^ = 0.37 (1)	0.540
Age class	0–3	7	12	19	36.8	1 (reference)		
	4–7	17	20	37	45.9	1.46 (0.48–4.70)		
	8–12	21	27	48	43.8	1.33 (0.45–4.14)		
	13–17	9	14	23	39.1	1.10 (0.31–3.95)		
	18–29	15	23	38	39.5	1.12 (0.36–3.60)		
	30–64	43	49	92	46.7	1.50 (0.55–4.36)		
	65+	7	6	13	53.9	2.00 (0.48–8.75)	LR χ^2^ = 1.79 (6)	0.938
**Bednet**	No	106	115	221	48.0	1 (reference)		
	Yes	13	36	49	26.5	0.39 (0.20–0.78)	FET	**0.007**
**Area**	Village	78	11	89	87.6	1 (reference)		
	Upland	39	102	141	27.7	0.05 (0.02–0.11)		
	Riverbanks	2	38	40	5.0	0.01 (0.001–0.03)	LR χ^2^ = 121.7 (2)	**<0.0001**
**Fowl**	No	62	51	113	54.9	1 (reference)		
	Yes	57	100	157	36.3	0.47 (0.29–0.77)	FET	**0.003**
**Dogs**	No	52	34	86	60.5	1 (reference)		
	Yes	67	117	184	36.4	0.37 (0.22–0.63)	FET	**0.0002**
Cats	No	95	111	206	46.1	1 (reference)		
	Yes	24	40	64	37.5	0.70 (0.39–1.25)	FET	0.251
**Pigs**	No	105	93	198	53.0	1 (reference)		
	Yes	14	58	72	19.4	0.21 (0.11–0.41)	FET	**<0.0001**
**Toilet/latrine**	Open	98	138	236	41.5	1 (reference)		
	Closed	21	13	34	61.8	2.27 (1.09–4.76)	FET	**0.041**
Waste disposal	Adequate	106	129	235	45.1	1 (reference)		
	Inadequate	13	22	35	37.1	0.72 (0.35–1.50)	FET	0.466
Crop-plot	No	14	14	28	50.0	1 (reference)		
	Yes	105	137	242	43.4	0.77 (0.33–1.68)	FET	0.550

⊕: seropositive; OR: unadjusted odds ratio; 95%CI: 95% confidence interval; d.f.: degrees of freedom; FET: Fisher's exact test; LR: likelihood-ratio test.

Variable names and p-values in **bold typeface** indicate covariates that entered the saturated model used as the starting point in backward step-wise regression; see text for details.

### Step-wise regression

A saturated, additive logistic model including all covariates for which bivariate NHT suggested association with seropositivity (bold typeface in Table 1) was then built as the starting point of backward SWR. The pre-established selection criterion/procedure resulted in the sequential exclusion of the following covariates: dog (LR χ^2^ = 0.006, p = 0.939), fowl (LR χ^2^ = 0.36, p = 0.851), closed toilet/latrine (LR χ^2^ = 2.04, p = 0.153), and pig (LR χ^2^ = 1.83, p = 0.177). The SWR ‘minimum adequate’ model therefore retained just two covariates: residence area within the settlement (LR χ^2^ = 131.31, p<0.0001), and regular bednet use (LR χ^2^ = 17.36, p<0.0001); this same model was selected by standard forward SWR (details not shown). This model (Table 2) suggests that village-like cluster residents were at much higher risk of MAYV infection, independent of bednet use, and that, conversely, regularly sleeping under a bednet protected from infection irrespective of residence area. The remaining covariates were considered irrelevant when adjusted for one another.

**Table 2:** Effect size estimates from the step-wise multivariate logistic regression ‘minimum adequate’ model.

Term	*β* (SE)	Adjusted OR* (95%CI)
Intercept	−1.30 (0.32)	-
Residence area		
Village	2.93 (0.38)**	1 (reference)
Upland	−0.49 (0.31)**	0.03 (0.01–0.08)
Riverbanks	−2.44 (0.51)**	0.005 (0.001–0.02)
Regular bednet use	−1.02 (0.28)	0.36 (0.21–0.62)

*β*: slope coefficient; SE: standard error; OR: odds ratio; 95%CI : 95% confidence interval.

* Odds ratios estimated as OR = exp(*β_i_*−*β*_Reference_);

** With respect to the other two area categories considered together.

### Multimodel inference

All model sets included a null model (estimating only the intercept), which represents the hypothesis of no predictable variation in seropositivity; as expected, this model always proved to be relatively very poor at explaining the data (Tables 3,4, and 5 and Table S1). Note that this null model is the same used in LR tests and in generalized *R* ^2^ calculations.

**Table 3:** Individual-covariate model set.

Model	AICc	ΔAICc	Likelihood	*wi*	*K*
Bednet	366.75	0	1	0.497	2
Age+Bednet	368.68	1.93	0.381	0.189	3
Sex+Bednet	368.70	1.95	0.377	0.188	3
Age+Sex+Bednet	370.66	3.91	0.142	0.070	4
Null	372.51	5.77	0.056	0.028	1
Age	374.17	7.42	0.024	0.012	2
Sex	374.43	7.69	0.021	0.011	2
Age+Sex	376.13	9.38	0.009	0.005	3

AICc: Akaike's Information Criterion corrected for small sample size; ΔAICc: variation in AICc relative to the best-performing model; Likelihood: likelihood of the model, given the data; *wi*: Akaike weights; *K*: number of estimable parameters.

**Table 4:** Household-covariate model set.

Model	AICc	ΔAICc	Likelihood	*wi*	*K*
Area+Crop	249.16	0	1	0.293	5
Area+Crop+Toilet/latrine	249.17	0.01	0.998	0.292	6
Area+Crop+Waste	250.01	0.85	0.655	0.192	6
Area+Crop+Toilet/latrine+Waste	250.06	0.90	0.640	0.187	7
Area	254.85	5.68	0.058	0.017	4
Area+Toilet/latrine	255.47	6.31	0.043	0.013	5
Area+Toilet/latrine+Waste	257.06	7.90	0.019	0.006	6
Toilet/latrine	369.63	120.47	0.000	0.000	2
Toilet/latrine+Waste	371.05	121.89	0.000	0.000	3
Crop+Toilet/latrine	371.52	122.36	0.000	0.000	3
Null	372.51	123.35	0.000	0.000	1
Crop+Toilet/latrine+Waste	373.02	123.86	0.000	0.000	4
Area+Waste	373.75	124.59	0.000	0.000	5
Waste	373.75	124.59	0.000	0.000	2
Crop	374.10	124.94	0.000	0.000	2
Crop+Waste	375.49	126.33	0.000	0.000	3

AICc: Akaike's Information Criterion corrected for small sample size; ΔAICc: variation in AICc relative to the best-performing model; Likelihood: likelihood of the model, given the data; *wi*: Akaike weights; *K*: number of estimable parameters.

**Table 5:** Domestic animal-covariate model set.

Model	AICc	ΔAICc	Likelihood	*wi*	*K*
Pig+Dog	346.38	0	1	0.371	3
Pig+Dog+Fowl	347.94	1.56	0.458	0.170	4
Pig+Dog+Cat	348.44	2.06	0.358	0.133	4
Pig	348.74	2.36	0.307	0.114	2
Pig+Fowl	349.51	3.13	0.209	0.077	3
Pig+Dog+Cat+Fowl	350.01	3.63	0.163	0.060	5
Pig+Cat	350.55	4.18	0.124	0.046	3
Pig+Cat+Fowl	351.54	5.16	0.076	0.028	4
Dog+Fowl	358.92	12.54	0.002	0.001	3
Dog	360.79	14.41	0.001	0.000	2
Dog+Cat+Fowl	360.98	14.60	0.001	0.000	4
Dog+Cat	362.69	16.32	0.000	0.000	3
Fowl	365.34	18.96	0.000	0.000	2
Cat+Fowl	367.23	20.86	0.000	0.000	3
Null	372.51	26.13	0.000	0.000	1
Cat	373.06	26.68	0.000	0.000	2

AICc: Akaike's Information Criterion corrected for small sample size; ΔAICc: variation in AICc relative to the best-performing model; Likelihood: likelihood of the model, given the data; *wi*: Akaike weights; *K*: number of estimable parameters.

The individual-covariate model set included seven models (Table 3); both the best-performing model and model-averaged effect-size estimates (details not shown) suggested a strong protective effect of regular bednet use; it was relatively much more important (*w* = 0.945) than age (*w* = 0.280) or gender (*w* = 0.270), whose effects were indistinguishable from zero. Therefore, only bednet use was retained for further analysis.

Next, we fitted all possible additive household-level models; this model set can be envisaged as representing hypotheses stating that in our study setting MAYV seropositivity simply varies among households with different traits, and comprises 15 models with all combinations of four candidate covariates (Table 4). All these covariates were retained for further assessment because they had relatively high *w* values: residence area (*w* = 1.000), crop-plot ownership (*w* = 0.965), closed toilet/latrine (*w* = 0.498), and, to a lesser extent, solid waste disposal (*w* = 0.385).

Third, we assessed all 15 possible model specifications including the four domestic animal covariates in our dataset (Table 5). On account of their relative importance, pigs (*w* = 0.999) and dogs (*w* = 0.735) were retained for testing, whereas cats (*w* = 0.267) and fowl (*w* = 0.337) were not considered any further.

**Figure 1:**
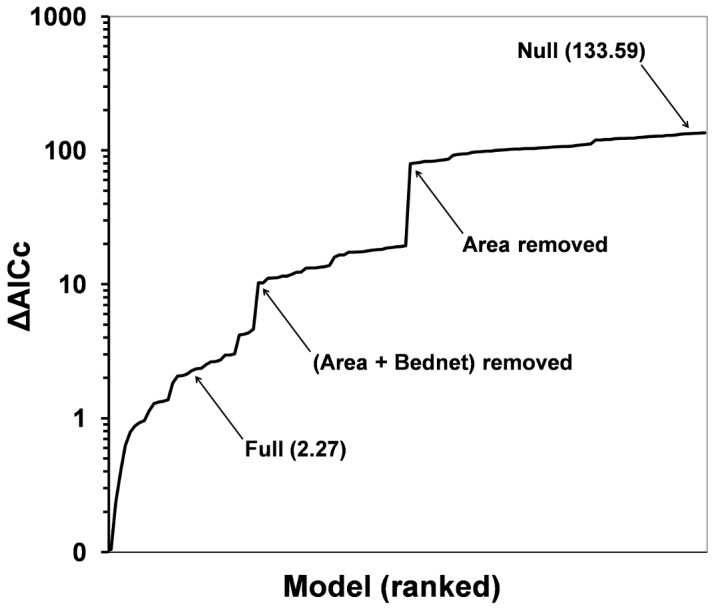
The models in the 128-model set used for inference on risk factors for Mayaro virus infection. Models were ranked according to variation in the Akaike's information criterion value of each model with respect to the best-performing model in the set (i.e., ranked by ΔAICc). Arrows highlight ΔAICc ‘leaps’ associated with the removal of the two most important covariates, residence area and bednet use. The position and ΔAICc value of the saturated model (Full) and the intercept-only model (Null) are also indicated. Note that the *y*-axis is in log_10_ scale.

Based on these results, we finally specified and compared the 128 models with all possible combinations of important individual-level (bednet use), household-level (residence area, crop-plot, toilet/latrine, waste disposal), and domestic animal covariates (pigs, dogs); the complete model set is provided in Table S1, and the subset of models with highest support from the data (ΔAICc<2) is presented in Table 6. Figure 1 illustrates variation in ΔAICc values across the 128 models in this set.

**Table 6:** Combined analysis: models with ΔAICc<2 in the 128-model set used for inference (see Table S1 for the complete model set).

Model	AICc	ΔAICc	Likelihood	*wi*	*K*
Area+Crop+Bednet	238.92	0	1	0.070	6
Area+Crop+Bednet+Toilet/latrine	239.03	0.10	0.949	0.067	7
Area+Crop+Bednet+Pig+Toilet/latrine	239.16	0.24	0.889	0.062	8
Area+Crop+Bednet+Pig	239.32	0.40	0.819	0.056	7
Area+Bednet*	239.55	0.62	0.732	0.051	5
Area+Crop+Bednet+Pig+Toilet/latrine+Waste	239.71	0.78	0.675	0.047	9
Area+Pig+Bednet	239.80	0.87	0.646	0.045	6
Area+Pig+Bednet+Toilet/latrine	239.85	0.92	0.630	0.044	7
Area+Bednet+Toilet/latrine	239.88	0.96	0.619	0.043	6
Area+Crop+Bednet+Pig+Waste	240.05	1.13	0.569	0.040	8
Area+Crop+Bednet+Dog+Toilet/latrine	240.21	1.29	0.526	0.037	8
Area+Crop+Bednet+Dog	240.24	1.32	0.517	0.036	7
Area+Crop+Bednet+Waste	240.26	1.33	0.513	0.036	7
Area+Crop+Bednet+Toilet/latrine+Waste	240.30	1.37	0.503	0.035	8
Area+Crop+Bednet+Dog+Pig+Toilet/latrine	240.75	1.83	0.401	0.028	9

AICc: Akaike's Information Criterion corrected for small sample size; ΔAICc: variation in AICc relative to the best-performing model; Likelihood: likelihood of the model, given the data; *wi*: Akaike weights; *K*: number of estimable parameters.

* The ‘minimum adequate’ model selected by step-wise regression analysis.

The best-performing model within this set had a generalized *R* ^2^ = 0.55 and a goodness-of-fit test χ^2^ = 10.21 (6 d.f., p = 0.116); the overall misclassification rate (model-predicted seropositivity status different from observed status) was just 0.19. These satisfactory fit metrics were similar for the rest of models in Table 6 (details not shown), and suggest that the models succeed in capturing important processes governing the relationships between covariates and the dependent variable. Adding interaction terms did not improve model performance or resulted in failure to reach convergence. Moreover, *a posteriori* addition of individual-level and domestic animal covariates removed in previous steps did neither improve the performance of any of the models with ΔAIC<2 nor change their relative positions (details not shown); therefore, we based inference on this 128-model set.

**Figure 2:**
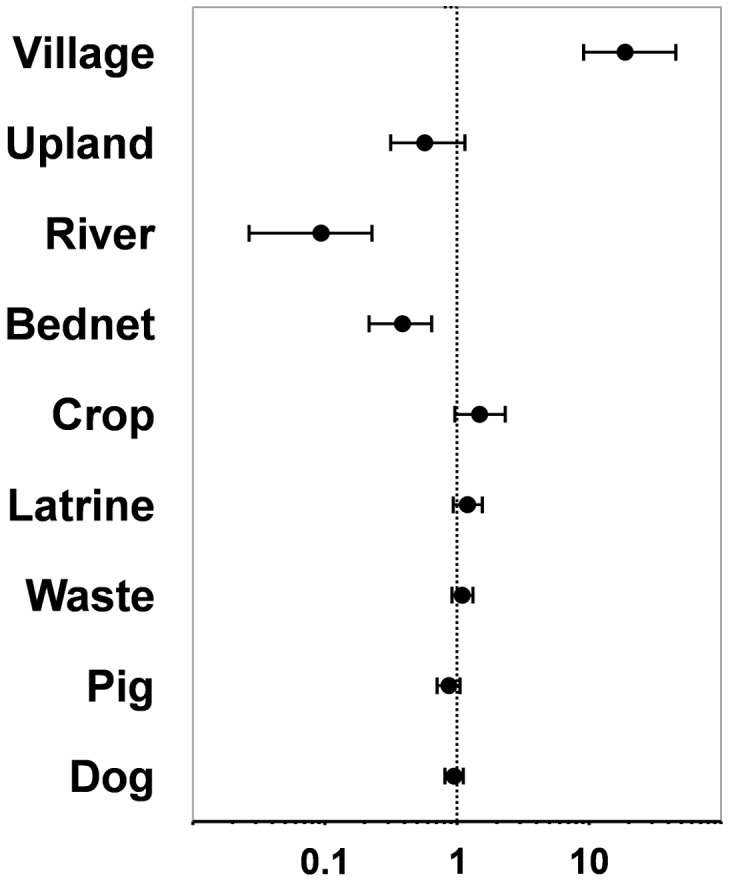
Effects of covariates on Mayaro virus seropositivity, averaged over the 128 models in the final set. Covariates describe: residence area (Village: village-like household clusters; Upland: upland areas; Riverbanks: better-preserved riverbank areas); bednet use (Bednet); crop-plot ownership (Crop-plot); use of a closed toilet/latrine (Toilet/latrine); adequate solid waste disposal (Waste); and the keeping/rearing of pigs (Pig) or dogs (Dog). Estimates are presented as odds ratios (solid circles) and 95% confidence intervals. The dotted line at odds ratio = 1 represents no effect; values >1 indicate a positive effect (increased risk of infection), and values <1 a negative (protective) effect.

When ranked according to their relative importance, the covariates considered in the final model set performed as follows: residence area, *w* = 1.000; regular bednet use, *w* = 0.997; crop-plot ownership, *w* = 0.627; closed toilet/latrine, *w* = 0.501; solid waste disposal, *w* = 0.364; pig, *w* = 0.351; and dog, *w* = 0.308. Table 7 presents the weighted average effect size over all models in the set (*β*s) for each of these covariates; exp(*β*) values, which estimate adjusted odds ratios, are presented in Figure 2. While MMI agreed with SWR in identifying residence area and no regular bednet use as strong independent predictors of MAYV seropositivity, it also showed that further covariates, and particularly crop-plot ownership, were important factors with non-negligible effects on infection risk. In sharp contrast with NHT results, domestic animals had little or no influence on MAYV seropositivity when adjusted for other covariates in our study setting.

**Table 7:** Model-averaged effect-sizes (*β* coefficients) from the final 128-model set.

Factor*	*β* coefficient	SE	Lower 95%CI	Upper 95%CI
Village**	2.93	0.41	2.21	3.82
Upland**	−0.56	0.33	−1.16	0.14
Riverbanks**	−2.37	0.55	−3.63	−1.48
Bednet use	−0.95	0.28	−1.53	−0.44
Crop-plot	0.39	0.22	−0.04	0.84
Toilet/latrine	0.19	0.13	−0.07	0.44
Waste	0.09	0.09	−0.09	0.28
Pig	−0.14	0.10	−0.35	0.05
Dog	−0.05	0.08	−0.21	0.11

SE: standard error; 95%CI: 95% confidence interval.

* See main text for the definition of covariates;

** With respect to the other two area categories considered together.

## Discussion

Arboviral infections are increasingly recognized as major public health threats. Geographic range expansions by dengue virus, West Nile virus, Japanese encephalitis virus, or, more recently, chikungunya virus have focused attention on their importance [2], [3]. Other arboviruses, however, have so far remained endemic to their putative areas of origin within developing countries, and this has perhaps contributed to their neglect [11]. Among the many viruses one could mention as examples, Rift Valley fever virus and O'nyong-nyong fever virus are of particular concern in Africa, and Venezuelan equine encephalitis virus and Mayaro virus in Latin America [3]. With effective vaccines unavailable (except for yellow fever), preventing infection heavily relies on vector control and personal protection measures, but both perform poorly. One key weakness of efforts in this direction is that robust risk factor analyses are lacking for most of these viruses; this limits our understanding of determinants of infection and, therefore, our ability to (i) identify risk situations/areas and (ii) design improved prevention strategies.

Here, we address this gap by presenting a case-study on MAYV; in addition to providing the first MMI-based assessment of risk factors for infection with this virus, we aimed at illustrating the caveats of conventional approaches often used to analyze observational data from cross-sectional surveys – and how MMI provides a powerful alternative. We discuss our results within the hypothetical framework outlined in the **Introduction**: if MAYV transmission is largely sylvatic, then spillover should increase infection risk mainly among people making frequent use of forested landscapes; those living in more degraded landscapes and those rarely entering forests, such as young children, should be relatively protected.

Prior to more detailed discussion, we consider several limitations of this study that must be kept in mind when interpreting our conclusions. First, even if EIA-ICC has been shown to be specific at detecting different anti-*Alphavirus* antibodies [45], some of our positive EIA-ICC results might be due to cross-reactions. We nonetheless tested all sera for anti-Venezuelan equine encephalitis virus antibodies and found just three putatively reactive samples, one of which was MAYV-negative. Thus, we feel confident that our serological results do reflect past MAYV infections, although we cannot completely exclude the possibility of a few cross-reactions with antibodies to closely-related but rarer viruses, particularly Una virus. In addition, we are unaware of any reliable data on the typical duration of anti-MAYV IgG; if high titers last long, this might weaken the relationships between seropositivity and (current) covariate values. Finally, we treated all subjects as independent, random samples of the settlement population; however, since several members of single families typically participated in the survey, the data are expected to present some degree of non-independence. This possibility, which has not been considered in previous MAYV risk factor analyses, could result in parameter variance underestimation [46], especially for household-level covariates. To check for possible effects of seropositivity clustering within households, we re-ran our ‘best’ model adding a term that indexed, for each subject, whether there were other seropositive individuals living in the same house; we found evidence of moderately increased risk (*β*_Other seropositive_ = 0.52±0.18; odds ratio 1.68, 95% confidence interval 1.18–2.39), but adjusted covariate effect estimates were very similar to those derived from MMI (cf. Table 7): *β*_Village_ = 2.76±0.42; *β*_Upland_ = −0.57±0.32; *β*_Riverbank_ = −2.18±0.53; *β*_Bednet_ = −0.85±0.29; and *β*_Crop-plot_ = 0.53±0.34. In addition, the overall biological plausibility of MMI results and the satisfactory model fit metrics both give us confidence that our results are a fair approximation to the data-generating processes [47].

We report an antibody prevalence in the middle-upper range of previous cross-sectional surveys [29], [33], [35], [48]–[53]. While, as throughout rural Amazonia, malaria is considered the main vector-borne disease in our study settlement, 75% of ∼1400 blood-smears from febrile patients seeking malaria diagnosis in 2004–2007 were *Plasmodium*-negative (SLBL and FAF, unpublished data). Even if some of these results are false-negatives, this suggests that pathogens other than *Plasmodium* are a major cause of acute febrile illness in the settlement. Our data, including the frequent mention by local residents of ‘joint pain’ as a typical feature of non-malarial febrile illness (unpublished observations from informal interviews), suggest that MAYV is likely involved in generating this epidemiological scenario.

But what factors modulate MAYV infection risk? Previous work is strongly suggestive of a pattern overtly dominated by forest transmission, with outbreaks sporadically recorded in rural communities embedded within rainforest environments [3], [4], [27]–[29], [33]–[37], [48]–[53]. Antibody prevalence is also typically higher, and clinical illness more frequent, among post-pubertal men, suggesting labor-related exposure involving forest activities [3], [4], [29], [51]; sex bias may nonetheless be absent in traditional communities in which both men and women make regular use of forested habitats [35], [48], [49]. All these conclusions are however based on merely descriptive accounts or on bivariate, NHT-based data treatments, rarely with age adjustment (e.g., [35]); they must therefore be interpreted with caution.

Our results are in partial contrast with these findings – and, hence, partly at odds with the prevailing hypothesis of forest transmission and sporadic spillover. Notably, age- and sex-specific seroprevalence suggest that people of both sexes and all age classes were similarly exposed to MAYV in our study population of non-indigenous settlers. The youngest seropositive subject was a girl just under two years of age residing in one of the village-like clusters; seropositive <3-year-olds were living in the two village-like clusters and in two distinct upland sites. This suggests, at least for these cases, that transmission was relatively recent and geographically widespread at the scale we consider, and casts doubts on the forest-transmission-only scenario.

In addition, our data strongly suggest that residing in the more densely populated village-like clusters greatly increased MAYV infection risk, whereas living in the better-preserved, sparsely populated riverbanks was protective. This sharply contrasts with previous reports explicitly showing the opposite pattern [29], [33]; again, it also contradicts the predictions of the forest-transmission hypothesis. However, our MMI approach revealed a role for crop-plot ownership as a relatively important risk factor, which would partially reconcile both results. Note that, had we based inference on NHT-SWR only, this relationship with forest crop-plots would have gone undetected and unreported (Table 1): the ‘best’ SWR model implicitly estimates [20] a zero effect for this covariate, even after adjustment, whereas MMI estimates a positive, marginally non-significant effect on the risk of MAYV seropositivity (Table 7, Figure 2). This crop-plot effect was in fact larger when young children (<8-years-old, *n* = 56) were excluded from the ‘best’ model in Table 6 (*β* = 0.74±0.40 *vs. β* = 0.54±0.33), suggesting that crop-plot work did increase risk-exposure.

The strong protective effect of regularly sleeping under a mosquito bednet (Figure 2) is also at odds with the assumption that MAYV is transmitted only by diurnal, canopy-dwelling mosquitoes. In addition, both NHT and MMI suggested an intriguing (albeit weak after adjustment) relationship between owning a closed toilet/latrine and an increased risk of seropositivity (Figure 2). Finally, and in plain disagreement with bivariate NHT results, domestic animals had very small effects (effectively not distinguishable from zero) on MAYV infection risk (Figure 2).

Thus, when considered as a whole, which MMI allows us to do, the effects of residence area and bednet use, and the clear lack of effect of age and gender, all suggest the possibility that MAYV cycles other than the classical ones (maintained by diurnal, forest-dwelling vectors) might occur in our study settlement. Ongoing research examines two main hypotheses: (i) that an alternative, nocturnal, endophilic vector species is involved in transmission, and (ii) that some local *Haemagogus* populations have shifted habitat and habits, adapting to densely populated landscapes and nocturnal biting. That MAYV can naturally infect *Psorophora* and *Mansonia* [54]–[56] and be transmitted by *Culex* and *Aedes* [57]–[59] lends more support to the first scenario. *Culex* and *Psorophora* were the most abundant vectors in the village-like clusters of our study site, where non-native *Aedes* spp. have never been recorded. *Haemagogus* and *Mansonia* seem to prefer forest and crop-plot habitats; both could therefore be involved in more typical MAYV transmission cycles in forested landscapes, which would help explain why owning a crop-plot increases risk, particularly among >8-year-olds.

### Conclusions

We have presented the first MMI-based assessment of risk factors for MAYV infection. The results suggest that two different, possibly overlapping MAYV transmission cycles might co-occur in a typical settlement of the Amazon agricultural frontier. If transmission by synanthropic vectors is confirmed, it could ultimately increase the risk of MAYV emergence in non-forest settings – perhaps even urban or periurban. This might have serious public health consequences [3], as the chikungunya example has shown [30], and calls for a tighter surveillance of arboviruses and their vectors in the Amazon. Finally, our analyses show how NHT and SWR result in the loss of valuable information when used to analyze observational data – a pervasive problem that is by no means particular to arbovirus epidemiology [12]–[14]. By revealing subtle associations that conventional risk factor analyses miss, MMI can foster our understanding of emerging infectious disease epidemiology and thus enhance disease control/surveillance systems.

## Supporting Information

Table S1 **The complete set of 128 models; covariates, AICc and related metrics, and number of parameters are given for each model.**
(XLS)

Text S1 **STROBE statement.**
(PDF)
